# The ethanol extract of *Periplaneta Americana* L. improves ulcerative colitis induced by a combination of chronic stress and TNBS in rats

**DOI:** 10.1590/acb370505

**Published:** 2022-08-12

**Authors:** Jing-na Zhang, Min-zhe Sun, Heng Liu, Han-chao Zhang, Huai Xiao, Yu Zhao, Chenggui Zhang, Hai-rong Zhao

**Affiliations:** 1 MM. Dali University – The First Affiliated Hospital – Genetic Testing Center – Yunnan, China.; 2 MM. Dali University – Yunnan Provincial Key Laboratory of Entomological Biopharmaceutical R&D – Yunnan, China.; 3 PhD. Dali University – Yunnan Provincial Key Laboratory of Entomological Biopharmaceutical R&D, and National-Local Joint Engineering Research Center of Entomoceutics – Yunnan, China.; 4 PhD. Dali University ( – Yunnan Provincial Key Laboratory of Entomological Biopharmaceutical R&D – Yunnan, China.; 5 PhD. Dali University – National-Local Joint Engineering Research Center of Entomoceutics – Yunnan, China.; 6 PhD. Dali University – The First Affiliated Hospital – Genetic Testing Center – and Yunnan Provincial Key Laboratory of Entomological Biopharmaceutical R&D – Yunnan, China.

**Keywords:** Periplaneta, Colitis, Ulcerative, T-Lymphocytes, Gastrointestinal Microbiome

## Abstract

**Purpose:**

To investigate the effects of Periplaneta americana L. on ulcerative colitis (UC) induced by a combination of chronic stress (CS) and 2,4,6-trinitrobenzene sulfonic acid enema (TNBS) in rats.

**Methods:**

The experiment UC model with CS was established in rats by a combination of chronic restraint stress, excess failure, improper, and TNBS. The body weight, disease activity index (DAI), colonic mucosal injury index (CMDI), histopathological score (HS) and pro-inflammatory mediators were measured. The content of corticotropin-releasing hormone (CRH) in hypothalamus or adrenocorticotropic hormone (ACTH) and corticosteroids (CORT) in plasma were evaluated by enzyme-linked immunosorbent assay. The proportion of T lymphocyte subsets was detected by flow cytometry, and gut microbiota was detected by 16S rDNA amplicon sequencing.

**Results:**

Weight loss, DAI, CMDI, HS and proinflammatory mediators were reversed in rats by *P. americana* L. treatment after UC with CS. Increased epidermal growth factor (EGF) was observed in *P. americana* L. groups. In addition, *P. americana* L. could reduce the content of CRH and ACTH and regulate the ratio of CD3^+^, CD3^+^CD8^+^ and CD3^+^CD4^+^CD25^+^/CD4^+^ in spleen. Comparably, *P. americana* L. changes composition of gut microbiota.

**Conclusions:**

The ethanol extract of *Periplaneta Americana* L. improves UC induced by a combination of CS and TNBS in rats.

## Introduction

Ulcerative colitis (UC) is an inflammatory intestinal disease (IBD) which was major involved in colon and rectum and closely related to immune system^1^^,^ 2. Although the etiology of IBD is vague and complicated, several contributory factors may influence disease activity, including smoking, poor diet, lack of physical exercise, host genetic factors, and a pro-inflammatory intestinal microbiome^3^. Studies of experimental chronic stress (CS) with dextran sulfate sodium (DSS)-induced UC in rats or mice have indicated that CS changes gut microbiota and drives inflammatory response^3^. CS drives the activation of the autonomic nervous system and the hypothalamus-pituitary adrenal (HPA) axis^4^^,^ 5, an increase in cortisol levels and proinflammatory cytokines such as interleukin (IL)-6, IL-1beta (β) and IL-8, tumor necrosis factor (TNF)-alpha (α)^5^. Interestingly, the composition of gut microbiota was dramatically changed after CS with colitis, with expansion of inflammation-promoting bacteria^3^^,^ 6 ^,^ 7.

*Periplaneta americana* L. with the dried or fresh adult for medicine^8^ is a traditional Chinese herbal medicine recorded in Shennong’s Herbal Classic. *P. americana* L. has widely used as a potential treatment of some diseases, such as cardiac failure^9^, hepatic fibrosis^10^, renal fibrosis^11^, osteoporosis^12^, gastric ulcer^13^, wound healing or burns^14^^-^ 18, cervical erosion, oral cavity ulcer, and UC^19^. In addition, drugs from *P. americana* L. such as Kangfuxin Solution (Z51021834) and Xinmailong Injection (Z20060443) are now extensively used for chronic heart failure and gastrointestinal ulcers and have been approved by the China Food and Drug Administration (cFDA), having achieved beneficial curative effects in clinical research.

Recent studies have indicated that *P. americana* L. ethanol extract has a potential effect for UC in rats by improving intestinal inflammation, ameliorating intestinal barrier function, and modulating composition of the intestinal flora, and reversing the intestinal-immune system^20^. However, the effect of *P. americana* L. on UC is induced by a combination of CS and 2,4,6-trinitrobenzene sulfonic acid(TNBS) enema in rats. Here, to investigate the effect of *P. americana* L. on CS with UC, we constructed CS with UC model in rats by a combination of chronic restraint stress, excess failure, improper diet, and TNBS enema. We confirmed that the ethanol extract of *P. americana* L. acts a novel therapeutic on CS with UC in rats.

## Methods

**Figure 1:**
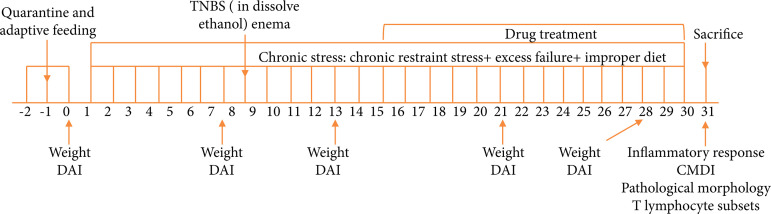
Experimental design.

Sprague-Dawley (SD) rats (male, 7-8 weeks) weighing 180~220 g were obtained from Hunan Leske Jingda Experimental Animal Co. Ltd. – License: SCXK (Xiang) 2016-0002 –, permit certificate for experiment: SYXK (Dian)-2018-0002. All the rats were housed in the experimental animal center of Dali University in a room at 20 ± 2°C with humidity 50% ± 2% in a 12-h light and dark cycle with free access to standard rat chow and tap water. All animals handling, including anesthesia, surgical procedures, post-operative care and sacrifice, has been approved by the Animal Care and Use Committee of Dali University, China. Timeline of experiment operation were illustrated schematically (Fig. 1).

### Reagents

The high-performance liquid chromatography (HPLC)-grade methanol was purchased from Thermo Fisher Scientific (Waltham, United States of America). The standard substance, including xanthine, hypoxanthine, inosine, uracil, and uridine, were purchased from National Institutes for Food and Drug Control (Beijing, China). TNBS (lot, No. SLBP0889V) was purchased from Sigma Company (United States of America). The enzyme-linked immunosorbent assay (ELISA) kits for inducible nitric oxide synthase (iNOS) (lot, No. 20161128), myeloperoxidase (MPO) (lot, No. 20161128), interleukin (IL)-17 (lot, No. 20161128), epidermal growth factor (EGF) (lot, No. 20161128), corticosteroids (CORT) (lot, No. 20161101), adrenocorticotropic hormone (ACTH) (lot, No. 20161101) and corticotropin-releasing hormone (CRH) (lot, No. 20161101) were provided by Nanjing Jiancheng Bioengineering Institute (Nanjing, China). Salazosulfapyridine (SASP) (lot, No. SLBP0889V), a sulfonamides antimicrobial drug, was purchased from Xinyi Shanghai Balance Pharmaceutical Co., Ltd. CD3-FITC (Cat#554832), CD4-PE (Cat#553730), CD8-APC (Cat# 553035), CD25-BV421 (Cat# 564571) and red blood cell lysis buffer (Cat# 349202) were selected from BD Biosciences.

### The ethanol extract of Periplaneta Americana L.

**Figure 2:**
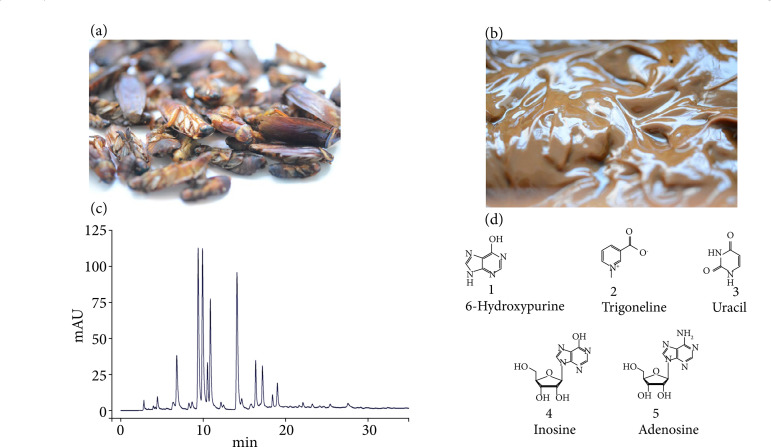
HPLC analysis of *Periplaneta americana* L., xanthine, hypoxanthine, inosine, uracil, and uridine. **(a)** The dried insect body of *P. americana* L. **(b)** Ethanol extract of *P. americana* L. **(c)** HPLC fingerprint chromatogram of *Periplaneta americana* L. **(d)** Peaks identified.

*P. americana* L. were provided by Good Doctor Pharmaceutical Group Co., Ltd. (Cheng du, China), and identified by Zizhong Yang (Yunnan Provincial Key Laboratory of Entomological Biopharmaceutical R&D). *P. americana* L. extract was performed by ethanol extraction. In detail, firstly, the powder of dry *P. americana* L. (200 g) (Fig. 2a) was extracted twice using 95% ethanol (1.2 kg, 0.8 kg, respectively) at 70°C for 12 h, and then the filtrates were mixed and condensed to 1.13-1.16 g/mL by rotary evaporation at 70°C. Secondly, purified water (v/v = 9:1) was added to the concentrated solution, heated and stirred at 70°C for 30 min, and then stood for 12 h. Finally, the oil in the upper layer was removed, and the solution in the lower layer was filtered and condensed to 1.20 g/mLby rotary evaporation at 70°C. Then, we acquired the ethanol extract of *P. Americana* L. used in animal experiments (Fig. 2b).

HPLC: high-performance liquid chromatography.

### Chemical characterization of Periplaneta americana L. extract

The content of peptide from the ethanol extract of *P. Americana* L. were measured using a lowry assay and the bovine serum albumin as a standard. Chromatographic analysis was performed on an Agilent 1260 series HPLC system equipped with a diode array detector, using a Sepax HP-C18 column (250 mm × 4.6 mm, 5 μm, 120A) at a column temperature of 35°C. The flow rate, the injection volume and detection wavelength were 0.8 mL/min, 5 μL and 254 nm, respectively. The solution A – 0.1% (v/v) acetic acid solution – and solution B (methanol) were utilized as the mobile phase, and the elution gradient was as it follows: 0-3 min (B 3%); 3-5 min (B 3% -8%); 5-10 min (B 8%-10%), 10-15 min (B 10%-35%), 15-30 min (B 35%-80%), 30-40 min (B 80%-3%), 40-45 min (B 3%). The *P. americana* L. extract (20 mg/mL) and standard compounds (0.5 mg/mL) were dissolved in 3% methanol water solution (v/v) and filtered via 0.22 μm membrane filter.

### Induced of chronic stress with ulcerative colitis in rats

CS (chronic restraint stress + excess failure + improper diet) was performed using published protocols^7^^,^ 21 ^-^ 23 with some modifications. Briefly, the rats were isolated in the individual cages and exposed to the following stressors:

- All rats were placed in water at 22 ± 1°C and swum for 10 min, continuously for 3 days;
- Restraint stress for 8 h (8h-16h) daily;
- Food deprivation for 24 h;
- Reversal of the light/dark cycle for 24 h.

These stressors were randomly performed once daily for 28 days.

Meanwhile, at the eighth days, rats were anesthetized with 10% chloral hydrate (300 mg/kg, intraperitoneally) and administered TNBS (7.5 mg/kg, in 30% ethanol) via rectal perfusion to imitate colitis^24^^,^ 25. The rats in normal group were administered with normal saline in the same way.

### Drug treatment

Sixty male SD rats were randomly divided into six groups (n=10):

- The normal group (i.g., normal saline, 20 mL/kg);
- CS+TNBS group (i.g., normal saline, 20 mL/kg);
- SASP, a sulfonamides antimicrobial drug group (i.g., 400 mg/kg);
- *P. americana* L. groups (i.g., 400 mg/kg).
- *P. americana* L. groups (i.g., 200 mg/kg).
- *P. americana* L. groups (i.g., 100 mg/kg).

At the 14th day after CS with UC, in addition to normal group and CS+TNBS group, rats of *P. americana* L. groups were continuously treated for 14 days. Moreover, normal group and CS+TNBS group were treated with normal saline.

### Determination of body weight and immune organ index

During the administration period, body weight was recorded at seven, 14, 21 or 28 days. After the rats were sacrificed by cervical displacement, the immune organs, including spleen and thymus, were collected and weighed immediately, and the immune organ index was calculated using Eq. 1:


Spleen or thymus index(mg/g)=weight of organ(mg)/weight of rat(g) (1)

### Disease activity index assay

During the experiment, body weights, stool, and body posture were monitored at seven, 14, 21 or 28 days to assess the disease activity index (DAI)^26^^-^ 28 in a blinded fashion. The DAI is the combined score of weight loss compared to initial weight, stool consistency, and body posture. The scores are evaluated as in Table 1.

**Table 1:** The scoring criteria of disease activity index*.

Score	Weight loss (%)	Stool consistency	Hematochezia status
0	no weight loss or gain	Normal and well-formed	Negative
1	1-5	Very soft and formed	Feces with occult blood (+)
2	6-10	Loose stool	Feces with occult blood (++)
3	11-15	Bloody stools (+)	Bloody feces (+)
4	> 15	Bloody stools (++)	Bloody feces (++)

* Total score: weight loss + stool consistency + hematochezia status.

### Morphological evaluation

Rats were sacrificed at the indicated time points, and colons were collected immediately for colonic length, colon mucosal damage index (CMDI), and histopathological score (HS).

The rats were anesthetized with pentobarbital sodium, and blood samples were removed from the abdominal aorta of rats to evaluate inflammatory cytokines. Then, the colon was dissected and quickly separated, and the length of colon was measured.

CMDI evaluation was performed as it follows^29^:

- 0: no damage;
- 1: mucosal congestion, edema, erosion or ulceration;
- 2: mucosal congestion, edema, mucous membrane rough, mild erosion or intestinal adhesion;
- 3: mucosal congestion, edema, moderate erosion and ulcer formation, but the diameter of ulcer was <1 cm;
- 4: mucosal congestion, edema, moderate erosion and ulcer formation, but the ulcer diameter was >1 cm.

### Histology analysis

Paraffin-embedded colon were cut into 5-μm sections and stained with hematoxylin & eosin (HE). As previously described^30^, histology was scored in a blinded fashion as a combination of inflammatory cell infiltration (score 0-4) and intestinal architecture damage (score 0-4) (Table 2). The total histologic score was derived by summing each individual score. Images were acquired with a microscope (DM2700 P, Leica).

**Table 2:** The criteria of histologic score.

Score	Architecture damage	Infiltration
0	No mucosal damage	The presence of occasional inflammatory cells in the lamina propria
1	Focal erosions	Increased numbers of inflammatory cells in the lamina propria
2	Slight crypt loss and focal ulcerations	Inflammatory cells extending into the mucosa and submucosa
3	Extended ulcerations and moderatecrypt loss	Inflammatory cells extending into the mucosa, submucosa, and sometimes transmural infiltration
4	Extensive crypt loss, mucosal damage,and extension into deeper structuresof the bowel wall	Severe transmural extension of the infiltrate

ELISA assay

To determine the content of CRH in hypothalamus, the rats were decapitated under anesthesia either on day 31. Then, the hippocampus was collected, weighed, and homogenized. Using normal saline as the homogenization medium, 10% tissue homogenate was prepared and centrifuged at 3,500 rpm for 15 min to acquire the supernatant, which was packed into the EP tube and kept at -80°C for future use. The content of CRH in hypothalamus or ACTH and CORT in plasma were detected by ELISA kits.

The blood was centrifuged at 3,000 rpm for 10 min at 4°C, and the supernatant was collected for the detection of IL-6, COX-2, iNOS using ELISA kit. In addition, colon (0.15) was homogenized with normal saline (1:9) and centrifuged at 3,000 rpm for 10 min. Subsequently, the supernatant was collected for the measurement of IL-17, EGF, and MPO by ELISA kits. According to manufacturer specifications, the levels of these cytokines were measured. The concentrations of cytokines were expressed as pg/mL.

### Flow cytometry

Flow cytometry was executed as our previous study described^31^. In brief, after the rats were sacrificed, to isolate T cells, spleens were collected under sterile conditions in ice-cold phosphate buffer saline (PBS) and filtered with sterile cell strainers (Biologix, 70 μm). Single-cell suspensions were prepared after the removal of red blood cells by ammonium-chloride-potassium (ACK) lysis buffer and centrifuged at 1,000 rpm for 5 min. Cell numbers were counted by a Coulter counter (Thermo Fisher). Cells were washed with buffer (PBS with 0.5% bovine serum albumin and 0.02% sodium azide) three times and subsequently stained with fluorochrome-conjugated monoclonal antibodies: CD3^+^-FIFC, CD4^+^-APC, CD8^+^-PE and CD25^+^-BV421. Samples were analyzed using Flow Cytometry (CytoFLEX S, Beckman CytoFlex S). Subsequent analysis was performed by FlowJo software (Tree Star Inc., San Carlos, CA, United States of America).

### 16SrRNA gene sequences of bacteria

The fecal samples in colon of rats were collected for the gut microbiota analysis (n=6 for every group). Total bacterial DNA was extracted from fecal samples of rats in each group according to instructions of DNA isolation kit (Tiangen Biotech Co., Ltd., Beijing) and subjected to cDNA synthesis by nucleic acid and protein analyzer (Thermo, United States of America, NanoDrop2000). Quantitative real-time polymerase chain reaction (RT-PCR) was performed using SYBR Green Supermix (Vazyme) according to the manufacturer’s instructions. The 16SrRNA gene sequences of bacteria were amplified by real-time fluorescence quantitative PCR (ABI, USA, StepOnePlusTMRT-PCR). The series of PCR primers for each flora were synthesized by Sangon Biotech Co., Ltd. (Beijing) (Table 3).

**Table 3:** Polymerase chain reaction primers.

Bacteria species	Target gene	Primers (5’ → 3’)
Total coliforms	16S rDNA	F:5’-TCCTACGGGAGGCAGCAGT-3’ R:5’-GGACTACCAGGGTATCTAATCCTGTT-3’
Bacteroides	16S rDNA	F: 5’-CTGAACCAGCCAAGTAGCG-3’ R: 5’-CCGCAAACTTTCACAACTGACTTA-3’
*Bifidobacterium*	16S rDNA	F: 5’-TCGCGTC(C/T)GGTGTGAAAG-3’ R: 5’-CCACATCCAGC(A/G)TCCAC-3’
*Lactobacillus*	16S rDNA	F: 5’-GTTAATACCTTTGCTCATTGA-3’ R: 5’-ACCAGGGTATCTTAATCCTGTT-3’
*Escherichia coli*	16S rDNA	F: 5’-AGCAGTAGGGAATCTTCCA-3’ R: 5’-CACCGCTACACATGGAG-3’
*Enterococci*	16S rDNA	F: 5’-CCCTTATTGTTAGTTGCCATCATT-3’ R: 5’-ACTCGTTGTACTTCCCATTGT-3’
Sulfate reducing bacteria	Alienation of sulfite reductase α subunit gene	F: 5’-CCA ACATGCACGGYTCCA-3’ R:5’-CGTCGAACTTGAACTTGAACTTGTAGG-3’

*Statistical analyses*

Statistical analyses were performed using GraphPad Prism 7 software. Kolmogorov-Smirnov test was used to determine the normal distribution of the samples. If the distribution of the sample was normal, we conducted statistical analyses in multiple groups using one-factor analysis of variance (ANOVA), followed by Dunnett’s test or Student’s t-test. If the samples were not distributed normally, a Kruskal-Wallis’ test was performed. A statistical difference was established when p < 0.05.

## Results

### Chemical characterization of Periplaneta americana L.

After the HPLC analysis of the chemical composition of *P. americana* L., five compounds were identified from *P. americana* L. They were respective inosine, 6-hypoxanthine, uracil, trigonelline, and adenosine (Figs. 2c and 2d). In addition, the percentage content of the five compounds in *P. americana* L. were determined – was 0.50, 0.31, 0.091, 0.065, and 0.064% (Fig. 2d) –, and the peptide content of *P. americana* L was 22.72%.

### Periplaneta americana L. treatment improves ulcerative colitis induced by a combination of chronic stress and TNBS in rats

**Figure 3:**
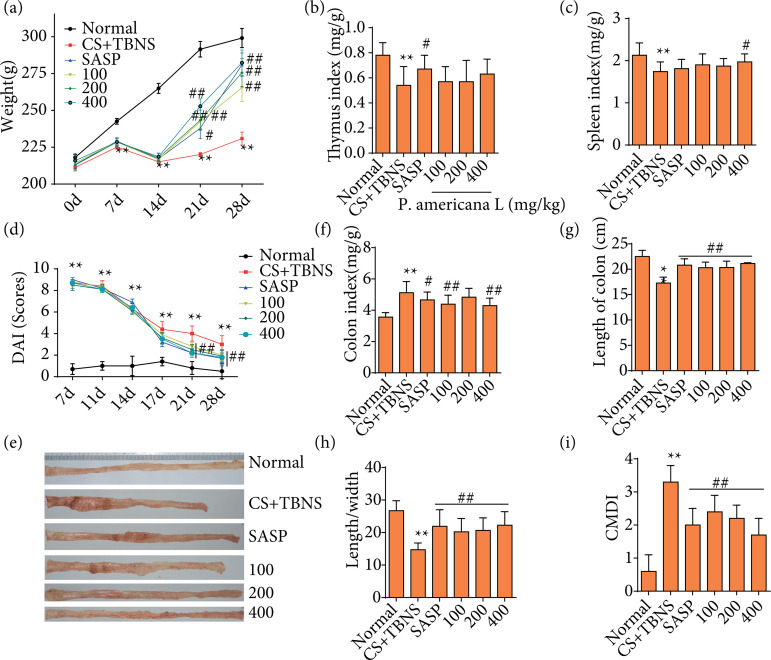
Effect of *Periplaneta americana* L. on weight loss, colonic length, colonic mucosal injury index in ulcerative colitis model induced by a combination of chronic stress and 2,4,6-trinitrobenzenesulfonic acid (TNBS). Rats continuously received intragastric of sulfasalopyridine or *P. americana* L. for 14 days. At the end of the experimental period, rats were sacrificed, and colons were removed. **(a)** Weight loss. **(b)** Thymus index. **(c)** Spleen index. **(d)** Disease activity index. **(e)** Colonic morphology. **(f)** Colonic index. **(g)** Length of the colon. **(h)** Length-width ratio of the colon. **(i)** Colonic mucosal injury index scores of each group were determined. Data were presented as mean ± standard deviation (n = 10 per group);

During the experiment, the weight of rats in the normal control group increased. At the seventh, 14th, 21st or 28th day, compared with the normal control group, the weight of rats in the CS with UC group decreased significantly (p < 0.01). At the 21st or 28th day, compared with the CS with UC group, the body weight of rats in the SASP group and *P. americana* L. groups increased remarkably (p < 0.01). The results are shown in Fig. 3a. Compared with the normal group, the thymus and spleen index (Figs. 3b and 3c) in the CS with UC group was significantly decreased, while the spleen index (Fig. 3c) was significantly increased in *P. americana* L. group (400 mg/kg, p < 0.05).

*,**Statistical significance between normal control versus other groups at p < 0.05 and p < 0.01 level; ^#,##^statistical significance between model control versus other groups at p < 0.05 and p < 0.01 level.

DAI is a convenient tool to measure the severity of colitis, and it was elevated in rats in each group at eight, 11, 14 or 17 days. However, reduced DAI in *P. americana* L. groups and SASP group were seen at 21 or 28 days as compared to the CS with UC group (p < 0.01) (Fig. 3d). As shown in Fig. 3e, no edema, ulcer and erosion were exhibited in the colon of normal group, whereas there were congestion, edema, multiple ulcer and erosion in the colon of CS with UC group, and most severely in the part of proximal anal end. As shown in Figs. 3f and 3i, it was evident that CS with UC gave rise to shortening of the colon length, decreasing in the ratio of colon length to width and elevating CMDI (p < 0.01). On the contrary, longer length of the colon and more mildly mucosal injury were seen in rats for CS with UC by *P. americana* L.-treated (p < 0.01). Similar effect was found in SASP group (p < 0.01). Taken together, these results confirmed that *P. americana* L. treatment improves UC induced by a combination of CS and TNBS.

### Periplaneta americana L. reduces release of proinflammatory cytokine, inhibits infiltration of inflammatory cell and damage of epithelial cell

Compared with the normal group, the release of IL-6, IL-17, iNOS and COX-2 were reduced in the CS with UC group (p < 0.01). However, decreased IL-6 or iNOS were observed in *P. americana* L. groups and SASP (p < 0.01). For IL-17 and COX-2,there was no significance between CS with UC group and *P. americana* L. groups. Recent data support the view that oxidative stress is also a formidable mediators of UC.

**Figure 4:**
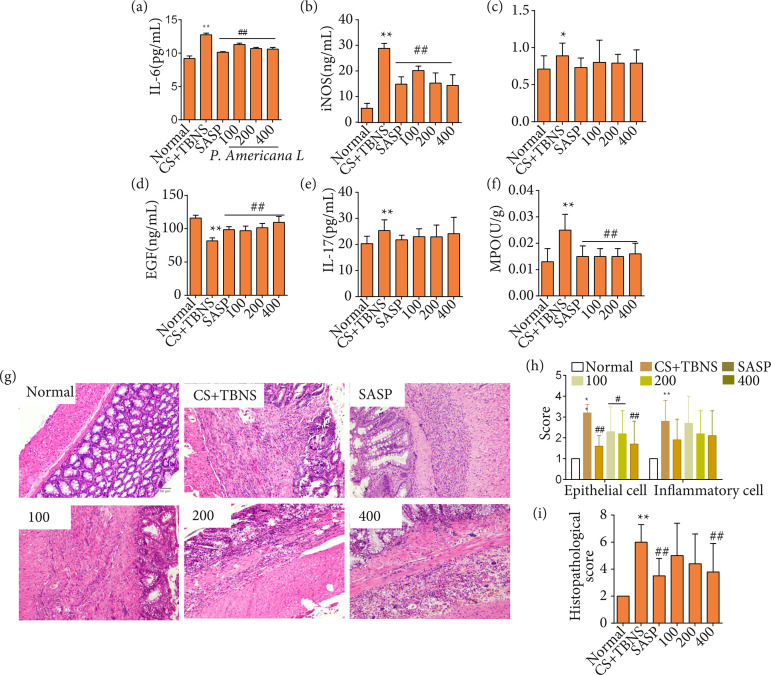
*Periplaneta americana* L. reduces release of proinflammatory cytokine, inhibits infiltration of inflammatory cell and damage of epithelial cell. These cytokines, such as **(a)** IL-6, **(b)** iNOS, **(c)** COX-2, **(d)** EGF, **(e)** IL-17, and **(f)** MPO were detected by ELISA. **(g)** The photomicrographs of hematoxylin & eosin staining on colonic sections of rats. **(h)** Epithelial cell scores and inflammatory cell infiltration were determined. **(i)** Histopathological scores of each group were determined. Scale bars: 50 μm; magnification: 200×. Data were presented as mean ± standard deviation (n = 6 per group).

IL: interleukin; iNOS: inducible nitric oxide synthase; COX-2: cyclooxygenase-2; EGF: epidermal growth factor; MPO: myeloperoxidase; ELISA: enzyme-linked immunosorbent assay.

Next, we measured the content of MPO. In comparison with normal group, significantly increased MPO was seen in CS with UC group. Comparably, *P. americana* L.-treated eased MPO level. The protective factors of intestinal mucosa, such as EGF, were associated into pathogenesis of UC. In this study, lower content of EGF in CS with UC group was surveyed as compared to the normal group (p < 0.01). Compared with CS with UC group, EGF level was elevated in a dose-dependent manner by *P. americana* L.-treated (Figs. 4a-4f).

As shown in Fig. 4g, HE staining revealed the structure of the submucosa, muscularis, and adventitia of the colonic mucosa was clear, the mucosa epithelium was integrated, and the goblet cells were clearly visible, there was no inflammatory cell infiltration in normal group. However, it was seen infiltration of inflammatory cells in mucosa and around the crypts that were polymorphonuclear leukocytes and lymphocytes in model group. Compared with the model group, these phenotypes were reversed in *P. americana* L. group.

On the other hand, colonic HS in CS with UC group was significantly higher (p < 0.01) as compared to normal group, whereas intragastric administration of drugs (SASP or *P. americana* L.) has demonstrated relief of inflammatory cell infiltration and injury of the mucosa epithelium (p < 0.01). The results are shown in Figs. 4g-4i. These data suggest that *P. americana* L. could reduce release of proinflammatory cytokine, inhibit infiltration of inflammatory cell and damage of epithelial cell.

### Periplaneta americana L. decreases the content of hypothalamus or adrenocorticotropic hormone and corticosteroids on ulcerative colitis induced by a combination of chronic stress and TNBS in rats

**Figure 5:**
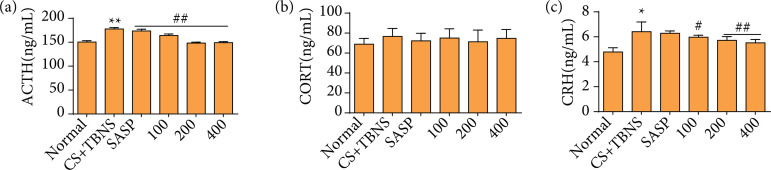
The effect of *Periplaneta americana* L. on the HPA axis in rats after chronic stress with ulcerative colitis. The level of **(a)** ACTH, **(b)** CORT, and **(c)** CRH was measured by ELISA.

HPA: hypothalamus-pituitary-adrenal; ACTH: adrenocorticotrophic hormone; CORT: cortisol; CRH: corticotropin releasing hormone; ELISA: enzyme-linked immunosorbent assay.

To assess whether the *P. americana* L. modulates the HPA axis, we examined the content of CRH, ACTH, and CORT. Compared with the normal group, the contents of ACTH, CRH, CORT in model group were significantly increased. Compared with the model group, the content of ACTH and CORT in the *P. americana* L. groups were significantly reduced (Figs. 5 a-5c).

### Periplaneta americana L. modulates CD4^+^/CD8^+^ ratio in ulcerative colitis induced by a combination of chronic stress and TNBS in rats

**Figure 6:**
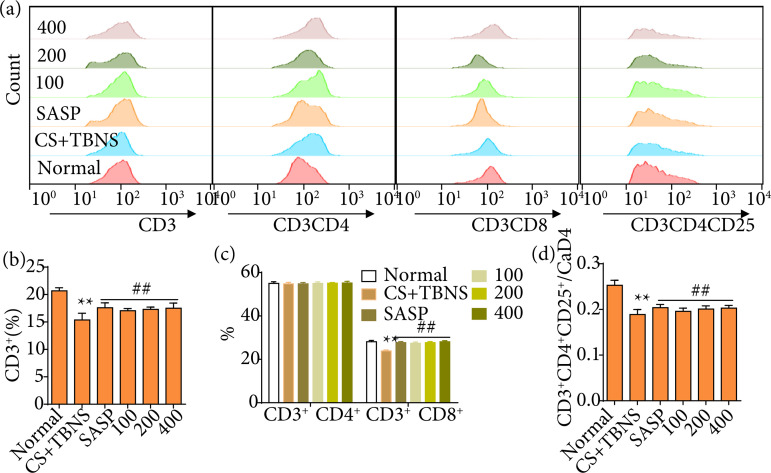
Effects of *Periplaneta americana* L. on T cell subtypes in the spleen in rats after chronic stress with ulcerative colitis. CD3^+^CD4^+^ T lymphocytes, CD3^+^CD8^+^ T lymphocytes, CD3^+^CD4^+^CD25^+^ regulatory T lymphocytes percentage were measured by flow cytometry. **(a)** Histogram of flow cytometry in each group; **(b)** CD3^+^ T lymphocytes; **(c)** CD3^+^CD4^+^ T lymphocytes and CD3^+^CD8^+^ T lymphocytes in spleen; **(d)** the ratio of CD3^+^CD4^+^ T lymphocytes to CD3^+^CD8^+^ T lymphocytes; **(e)** the ratio of CD3^+^CD4^+^CD25^+^ regulatory T lymphocytes to CD3^+^CD4^+^ T lymphocytes. Data were presented as mean ± standard deviation (n = 4 per group).

As compared to the normal group, CD3^+^ T lymphocytes was decreased in CS with UC rats, which was reversed by *P. americana* L. or SASP treatment (p < 0.01). There was no significance in each group for CD3^+^CD4^+^ T lymphocytes, but reduced CD3^+^CD8^+^ T lymphocytes were seen in CS with UC rats. Comparably, *P. americana* L. or SASP-treated could elevated CD3^+^CD8^+^ T lymphocytes (p < 0.01). Simultaneously, risen CD3^+^CD4^+^/CD3^+^CD8^+^ ratio was exhibited in model group, while this phenotype was changed after administration of *P. americana* L. In addition, CD3^+^CD4^+^CD25^+^/CD4^+^ ratio of T lymphocytes were declined in CS with UC rats as compared to the normal group. In comparison to CS with UC group, markedly increased CD3^+^CD4^+^CD25^+^/CD4^+^ ratio of T lymphocytes were displayed in *P. americana* L. groups (p<0.01). The results are shown in Figs. 6a-6d.

### Periplaneta americana L. changes composition of gut microbiota in rats after chronic stress with ulcerative colitis

Gut microbiota was exhibited as a key factor in the function and development of the immune system, and study has linked the gut microbiome for CS with TNBS. As shown in Table 4, for total coliforms, it was not significant in each group. At the genus levels, the composition of microbiota all changed in CS+TNBS-treated rats. Comparably, *P*. *americana* L. changed composition of gut microbiota, such as *Bacteroides*, *Lactobacillus*, and *Escherichia coli*.

**Table 4:** The effect of *Periplaneta americana* L. on gut microbiota in rats after chronicstress with ulcerative colitis (Lg quantity mean).

Groups	The types of intestinal flora						
	Total coliforms	*Bacteroides*	*Bifidobacterium*	*Lactobacillus*	*Escherichia coli*	*Enterococci*	*Sulfate reducing* *bacteria*
Normal	8.60±0.22	7.51±0.13	6.18±0.31	6.65±0.15	5.25±0.38	5.12±0.71	5.51±0.75
CS+TNBS	8.62±0.04	7.37±0.04*	5.57±0.14*	6.67±0.15	5.51±0.86	4.41±0.52*	5.38±0.29
SASP	8.44±0.17	7.28±0.28	5.37±0.52	6.44±0.28	4.65±0.24	3.78±0.23*	3.90±1.04*
100	8.52±0.37	7.38±0.44	5.96±0.42	6.42±0.51	5.97±0.47	4.57±0.66	5.28±0.57
200	8.43±0.15	7.41±0.21	6.13±0.46#	6.64±0.60	4.78±0.27#	3.82±0.40*	5.09±0.14
400	8.46±0.25	7.52±0.46#	5.39±0.24	6.35±0.63	5.25±0.88	3.86±0.39*	5.18±0.14

CS: chronic stress; TNBS: 2,4,6-trinitrobenzenesulfonic acid; SASP: salazosulfapyridine;

* p < 0.05; ^**^p < 0.01 *vs*. the normal group;

# p < 0.05; ^##^p < 0.01 *vs*. the CS group (n = 6).

## Discussion

UC is a complex, chronic, immune-mediated IBD, and it is clinically characterized by weight loss, abdominal cramping, pain, and diarrhea^32^. The stressful modern lifestyle also results in UC. With life rhythm speed, increasingly with the social competition, and the heavily mental pressure, CS will contribute a lot to anxiety, insomnia, depression, and even mental disorder. In addition, CS promotes the progression of diseases such as cancer and IBD. This study shows that *P. americana* L. could decrease the susceptibility of rats to CS with TNBS-induced UC. Before *P. americana* L. treatment, we detected the chemical characterization of *P. americana* L. Five compounds were identified from *P. americana* L. They were respective inosine, 6-hypoxanthine, uracil, trigonelline and adenosine. We observed that CS aggravated progress of TNBS-induced UC in rats, which were reversed by *P. americana* L.-treated after CS with UC.

The key role of innate myeloid cells in UC is reflected by the strong pro-inflammatory effects of the cytokines that they release, particularly IL-6 and TNF-?^33^. IL-6 is elevated in the inflamed intestinal mucosa, and suppression of IL-6 signaling relieved colitis in mouse models and also had beneficial effects in a clinical trial of patients with Crohn’s disease^34^. Here described observation, *P. americana* L. inhibits release of pro-inflammatory cytokine (IL-6) that are typically upregulated in UC, as well as iNOS, which produces nitric oxide and is associated into the pathogenesis of colitis^35^. Significantly decreased iNOS was seen by *P. americana* L.-treated. Expression of inflammatory proteins which include COX-2 and iNOS are believed to play a key role in UC^36^^,^ 37. Previous study has shown that phenolic compounds from *P. americana* L. with potent COX-2 inhibitory activity^38^. However, the activity of COX-2 was not suppressed in this study even though increased COX-2 was observed in rats of CS with UC. Concurrently, this was supported by pathological observation, following *P. americana* L. treatment, mucosal injury, including ulcers, edema and mucosa hyperemia, or bleeding were gradually ameliorated.

EGF is a potent mitogenic peptide produced by salivary glands. EGF enemas are an effective treatment for UC^39^. Increased EGF was observed in *P. americana* L. groups. *P. americana* L. promotes EGF secretion, found in wound repair and regeneration^14^. According to reports, CS with UC may involve in the abnormalities of multi-system functions, including modern endocrine, digestion, immunity, and nerve, of which HPA axis changes are particularly prominent. When stress-induced corticosteroid secretion, however, normal activity in the HPA is not inhibited and may even be augmented. Experiments in rats have shown that stress also induces facilitation of subsequent activity in the HPA axis, and stress signals can stimulate the secretion of CRH to increase and cause pituitary ACTH to increase, eventually leading to an increase of CORT. The excessive increase of CORT can also damage the negative feedback regulation mechanism of HPA, resulting in sustained hyperfunction of HPA, thus causing disorders of multiple systems, such as organism, nerve, endocrine and immunity. The contents of CRH in the hypothalamus, ACTH in plasma and CORT in the *P. americana* L. group were significantly reduced.

Moreover, *P. americana* L. regulates the ratio of CD3^+^, CD3^+^CD8^+^ and CD3^+^CD4^+^CD25^+^/ CD4^+^ in spleen. Abnormal regulation of the immune system is involved in the final process of the pathogenesis of UC. In the mucosa of patients with UC, the homoeostatic balance between regulatory and effector T-cells (e.g., T-helper [Th] 1, Th2, and Th17) is disturbed^40^^,^ 41. It is considered that UC is an atypical Th2 type immune response disease, including T cells, cytokines, or other cell receptors, which leads to the abnormality of inflammatory cells and inflammatory mediators, or intestinal mucosal tissue damage. T lymphocytes are a core link of antigen presentation, including CD4^+^ (Th) and CD8^+^ (Ts/Tc). The dynamic balance of the CD4^+^/CD8^+^ ratio determines the state of immune regulation and immunity, and a decrease in the ratio of CD4^+^/CD8^+^ marks the inhibition of immune function. CD4^+^ T cells can differentiate into CD4^+^CD25^+^ regulatory T cells (Treg) under cytokines and environmental effects. Treg cells are pivotal for the regulation of intestinal inflammation. Treg cells oppress the responses of defector T cells, such as Th1 and Th17 cells, and play an important protective role in mice with UC.

The results of this study suggest that the immune regulation of rats in the model control group is out of balance, showing that the proportion of CD3^+^ T cells and CD3^+^CD8^+^ T cells and the ratio of CD4^+^CD25^+^/CD4^+^ are significantly decreased, while the ratio of CD4^+^/CD8^+^ is significantly higher than the normal group. After treated with *P*. americana extract and SASP, the proportion of CD3^+^ T cells and CD3^+^CD8^+^ T cells, and the ratio of CD3^+^CD4^+^CD25^+^/CD4^+^ increased significantly, while the ratio of CD4^+^/CD8^+^ decreased significantly (p < 0.01), suggesting that *P. americana* L. extract has immunomodulatory effect.

The gut-microbiota-brain axis is implicated an intricate system of bidirectional communication between the central nervous system and the gastrointestinal tract that principally includes immune mediators, the vagus nerve, gut microbiota-derived metabolic pathways, and neuroendocrine pathways. Our results indicate *P*. *americana* L. changes composition of gut microbiota in rats after CS with UC. Comparably, *P. americana* L. changes composition of gut microbiota.

## Conclusion

*P. americana* L. extract has a therapeutic effect on UC in rats with CS. Its therapeutic effect may be related to relieving the persistent hyperactivity of HPA axis, increasing the ratio of CD3^+^CD4^+^CD25^+^/CD4^+^ and reducing the ratio of CD4^+^/CD8^+^ to restore the balance of immune regulation, changing composition of gut microbiota.
